# Qualitative modelling of social determinants of health using group model building: the case of debt, poverty, and health

**DOI:** 10.1186/s12939-022-01676-7

**Published:** 2022-05-19

**Authors:** Laurens Reumers, Marleen Bekker, Henk Hilderink, Maria Jansen, Jan-Kees Helderman, Dirk Ruwaard

**Affiliations:** 1 Department of Health Services Research, Care and Public Health Research Institute (CAPHRI), Maastricht University, Maastricht, The Netherlands; 2 Chair group Health and Society, Center for Space, Place and Society, Wageningen University and Research, Wageningen, The Netherlands; 3 National Institute for Public Health and the Environment, Bilthoven, The Netherlands; 4 Academic Collaborative Center for Public Health, Public Health Service South Limburg, Heerlen, The Netherlands; 5 Institute for Management Research, Radboud University, Nijmegen, The Netherlands

**Keywords:** Social determinants of health, Wicked problems, Group model building, System dynamics, Methodology, Health impact assessment, Poverty, Debt

## Abstract

**Background:**

Social determinants of health (SDoH) are known to have a large impact on health outcomes, but their effects are difficult to make visible. They are part of complex systems of variables largely indirect effects on multiple levels, constituting so-called wicked problems. This study describes a participatory approach using group model building (GMB) with stakeholders, in order to develop a qualitative causal model of the health effects of SDoH, taking poverty and debt in the Dutch city of Utrecht as a case study.

**Methods:**

With GMB we utilised the perspective of stakeholders who are directly involved in policy and practice regarding poverty, debt, and/or health. This was done using system dynamic modelling, in three interactive sessions lasting three hours each. In these sessions, they constructed a model, resulting in a system of variables with causal relationships and feedback loops. Subsequently, the results of these GMB sessions were compared to scientific literature and reviewed by a panel of researchers with extensive experience in relevant scientific fields.

**Results:**

The resulting model contains 71 causal relationships between 39 variables, 29 of which are present in feedback loops. The variables of *participation in society*, *stress*, *shame*, *social contacts* and *use of services/provisions* appear to hold prominent roles in the model’s mechanisms. Most of the relationships in the model are supported by scientific literature. The researchers reviewing the model in the scientific meeting agreed that the vast majority of relationships would concur with scientific knowledge, but that the model constructed by the stakeholders consists mostly of individual-level factors, while important conditions usually relate to systemic variables.

**Conclusions:**

Building a model with GMB helps grasp the complex situation of a wicked problem, for which it is unlikely that its interrelationships result in a fully intuitive understanding with linear mechanisms. Using this approach, effects of SDoH can be made visible and the body of evidence expanded. Importantly, it elicits stakeholders’ perspectives on a complex reality and offers a non-arbitrary way of formulating the model structure. This qualitative model is also well suited to serve as conceptual input for a quantitative model, which can be used to test and estimate the relationships.

**Supplementary Information:**

The online version contains supplementary material available at 10.1186/s12939-022-01676-7.

## Background

### Social determinants of health and health outcomes

There is evidence that suggests that social determinants of health are responsible for at least about half of a population’s health loss [1, 2]. Social determinants of health are non-medical factors that affect health outcomes. According to a definition given by the World Health Organization, SDoH can be regarded as “the conditions in which people are born, grow, work, live, and age, and the wider set of forces and systems shaping the conditions of daily life”, including “economic policies and systems, development agendas, social norms, social policies and political systems “[3]. These can, according to the rainbow model by Dahlgren and Whitehead [4], be categorised in individual lifestyle factors, social and community networks, living and working conditions, and eventually to general socioeconomic, cultural and environmental conditions. The further one goes towards the final and outer layer, the more distal and so less proximal, i.e., individual, determinants become.

Estimating health outcomes related to the social determinants of health is a difficult task. These outcomes are often related to a mix of both distal and proximal determinants [1, 5]. As such, they are part of complex systems, consisting of determinants and relationships between determinants on multiple levels that are usually not linear nor unidirectional [6]. As a rule, these systems are constituted by many indirect relationships between a great number of interrelated variables and it is generally not immediately apparent which variables are relevant and which are not. This is why the whole of all layers of social determinants of health can be considered to constitute so-called wicked problems. Wicked problems are issues that, in short, have many stakeholders but not one definitive shared problem definition, are unique, and do not have clear boundaries [7, 8]. The perception of a wicked problem itself varies between different stakeholders and there is no test to ascertain, either prospectively or retrospectively, whether the ‘right’ solution will be or has been implemented. Trying to tackle a wicked problem with a rational-technical approach will ignore the values and experiences of stakeholders and therefore the problem itself [8], likely resulting in a solution that attempts to fix the wrong issue with the wrong tools. A suitable way of approaching such wicked problems is deploying participatory methods combined with systems science, in which the knowledge of stakeholders, who are experts in experience, is used in order to capture the complexity of the system in a particular context [9]. Similar approaches have been used in several previous studies on social determinants of health [10–17]. The use of participatory methods in conjunction with systems science allowed these studies to model systems of interrelated variables in a non-arbitrary manner and it allows unrecorded knowledge to be used in their formulation [18].

### Group model building

Group model building (GMB) is a method that facilitates stakeholder participation in order to construct a qualitative conceptual model of a particular issue, using system dynamics modelling. Its purpose is to collect information from a group of stakeholders in order to increase understanding of complex problems [19, 20]. In this paper, we describe how this method can be used to capture relationships between social determinants of health and health outcomes. We illustrate this by taking a case in which two related social determinants of health are studied: poverty and debt. In this way, we aim to make tangible complex interrelationships and get a handle on the wicked problem by using the perspective of stakeholders who are directly involved – as professionals and/or as affected citizens – with the issue in policy and practice. This is intended to better understand the problem itself and hopefully give an indication of which kinds of policy options may be expected to be effective and appropriate.

### Poverty, debt and health outcomes

Poverty and debt are seen as important [21] and broad social determinants of health [1, 4], which affect health indirectly through material, behavioural and psychosocial factors [22–26]. While there is strong evidence for the existence of associations between poverty, debt, and health, it is less clear how exactly these variables are related to each other. The variables of poverty and debt are included as two separate determinants in this study. Although they are logically related, debt is often not included in definitions of poverty and can be seen as distinct. Poverty and debt are regarded as (social) determinants of health, but the reverse effect is just as likely to occur: health is also expected to affect poverty and debt [27–29]. Such feedback processes are typical for social determinants of health and necessitate an approach using systems science [6].

### The stakeholders’ perspective

This study describes how the complex relationships between social determinants of health – in this case poverty and debt – and health are understood and experienced by stakeholders directly involved in policy and practice. The stakeholders hold experiential knowledge of the local context of the case described in this paper, which is important for gaining insight into the wicked problem and eventually for acceptable, feasible and useful solutions. They use their knowledge to specify a complex system of relationships between a number of relevant variables in detail. Merely concluding that the variables are associated is not specific enough; it is also necessary to make clear *how* causal mechanisms seem to work and therefore why certain outcomes are expected from the stakeholders’ perspective. At the same time, both the effects of poverty and debt on health and the effects of health on poverty and debt will have to be included.

### Research question

The research question of this paper is a methodological one and can be formulated as: *How can group model building contribute to gaining insight into a wicked problem concerning social determinants of health?* In order to address this question and demonstrate the methodological approach in practice, the stakeholders’ views on the interrelationships between poverty, debt, and health are made explicit and strengthened by carrying out validation checks.

## Methods

### Setting

This study focused on the case of poverty and debt in the Dutch city of Utrecht (hereafter ‘Utrecht’ shall be used in this paper to mean ‘the city of Utrecht’). Utrecht had approximately 360,000 inhabitants in 2021 [30] and has well-established poverty reduction efforts and a sizeable number of organisations from civil society involved with combating poverty.

In 2018, over 15% of households in Utrecht – approximately 27,700 households – were reported as having a low income, defined as having 125% or less of the legal social minimum income, and almost two-thirds of this group had an income of below 105% of this minimum [31]. This legal social minimum depends on household composition; for example, for an adult living alone this is 70% of the legal minimum wage. In January 2018, this was €992 gross per month [32, 33]. In a survey conducted by the municipality in 2018, 6% of all respondents stated that they had trouble or a lot of trouble to make ends meet [34]. Almost half of this group was not able to fully participate in society. Of all respondents in the study, 7% indicated that they have to use their savings in order to make ends meet and 4% is forced to create debts [35]. The percentage of inhabitants of Utrecht who have problematic debts is estimated to be somewhere between 7 and 20% [34, 36].

### The group model building (GMB) process with stakeholders

First, relevant stakeholders were identified by purposeful selection. This was done in co-operation with policy advisors employed by the municipality, who had already been working on the issue and therefore possessed long-established professional connections to a heterogeneous group of stakeholders in the field. The selected stakeholders have experience with and are embedded in the practical side of the issue. Their experiences with and views of the topic at hand are usually not quantified or quantifiable, but hold a wealth of information and tacit knowledge about persons in poverty [18]. Short, semi-structured interviews were held in order to gauge the stakeholders’ experience, views on the issues, and expectations for the sessions. The interview guide can be found in Additional file 1. The input obtained from these interviews was used for optimising the outline of the stakeholder sessions.

Three stakeholder sessions took place in three consecutive weeks lasting approximately three hours each; these had the aim of constructing a causal model of how poverty, debt and health relate to each other in Utrecht. The sessions were facilitated by LR and MB, who guided the participants through the GMB process using the Vensim software package [37] to build a qualitative model. The participants were the ones who would decide what the model structure would look like. In each session, twelve to fourteen stakeholders participated – mostly the same participants in every session. In total, there were sixteen different individuals taking part. Six of them worked at community organisations, three in healthcare, three in a policy-making position at the municipality, two in debt assistance and two at interorganisational co-operation organisations. Some of the participants had also dealt with poverty and debt in their personal lives.

In the first GMB session, its general topic – the interrelationships between poverty, debt, and health – was first briefly introduced. These three variables were not presented in terms of determinant and outcome, as the participants were not supposed to only think about how poverty and debt may affect health, but also how health may in turn affect poverty and debt. After a short introduction of the method and the aim of the sessions, the participants were given their first assignment, which was based on the often-used “hopes and fears” GMB script [38]. In this assignment, they were asked to individually write down (and if possible, draw graphs of) three variables they thought were central outcome variables to be addressed concerning the topic. In other words: what are the main intrinsic problems in society concerning poverty, debt, and health that they would like to see improved, ideally? This exercise was meant to help participants agree amongst themselves on what the focus of the GMB sessions should be. The variables that the participants thought as being most central to the issue were listed and subsequently subjected to a voting round. This led to the stakeholders selecting four ‘central variables’, which would serve as a starting point for later modelling exercises. Finally, all other variables that the participants had thus far identified as important to the topic were ordered in a visual way, by placing them near the central variable to which they were most closely related.

The second session focused on building the first parts of the causal loop diagram. This was done by connecting the other variables listed in the first session (that had not been selected to be central variables themselves) to the closest one of the four selected central variables. In this way, four small, separate models were constructed (see Additional file 2 for the results of several modelling steps from sessions 2 and 3). In the second part of this session, the participants worked to identify links between the four different models, in order to combine the four small models into one large model. The central variables were not to be connected to each other directly, but needed at least one variable in between. This variable in between, the participants could add to the model if it was not already present. The reason for requiring at least one variable between central variables was that the participants were thus compelled to formulate mechanisms that explain *how* these relationships work.

In the third and final session, the facilitators gave a short introduction on the difference between direct, individual, proximal determinants and more indirect, collective, distal, structural determinants. Then the participants, working in small groups, were asked to identify such structural determinants relevant to their model and to add and connect them to the appropriate part or parts of the model. They were subsequently asked to also add any important variables that were not yet present in the model. With the conclusion of this part of the session, the stakeholder model was completed. This model can be found in the results section of this article (Fig. 3). The final part of the third session was used to show the participants how they could use the model themselves, using several examples from local practice that they proposed. This exercise elaborated on how an external activity can tie into the model structure and how its expected indirect effects can be traced. After completing the final session, short evaluation forms, asking about experiences and satisfaction with the GMB sessions, were given to the participants to fill out. A summary of the qualitative results of the study – its implications and the models – was also given to the stakeholders afterwards.

**Fig. 1:**
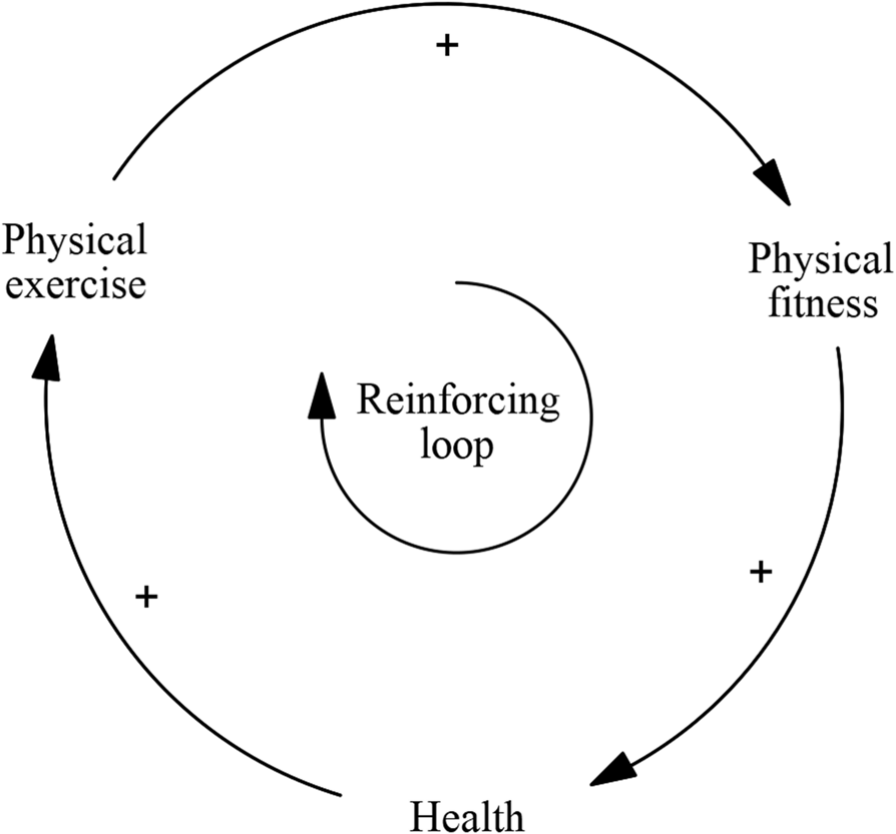
An example of a reinforcing feedback loop (Vensim, PLE version 9.0.1)

**Fig. 2:**
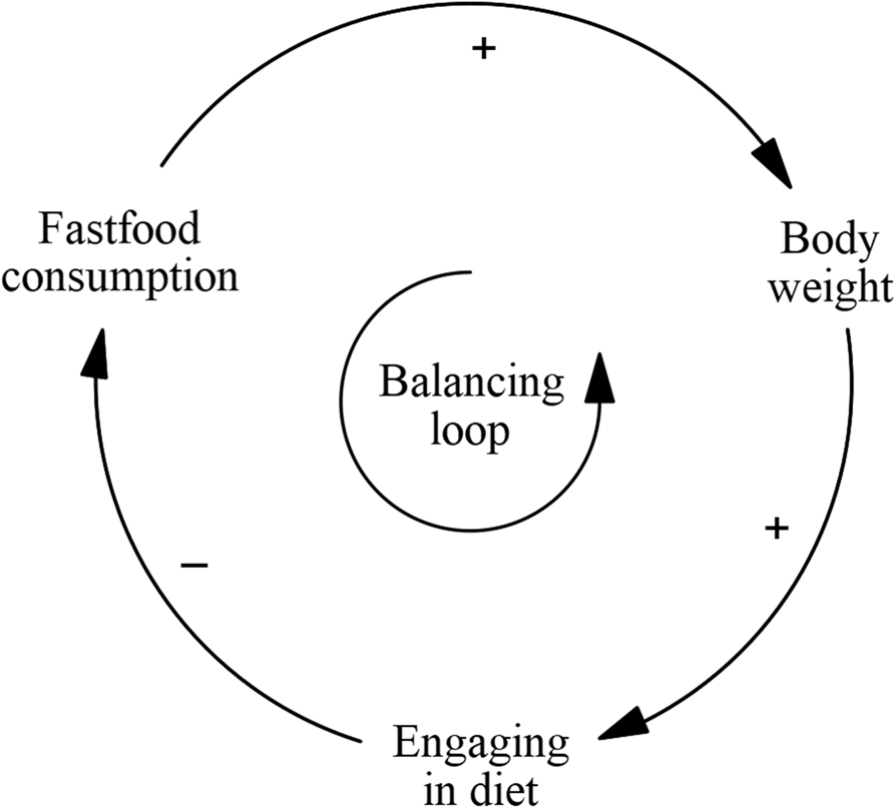
An example of a balancing feedback loop (Vensim, PLE version 9.0.1)

The sessions resulted in a causal loop diagram (CLD), which is a qualitative model that is used in system dynamics modelling, that consists of variables and causal arrows that form feedback loops. System dynamics modelling works with aggregated-level logic, which the facilitators made sure was maintained in the GMB sessions. A feedback loop exists when two or more variables directly or indirectly influence each other. For example, physical exercise can increase the level of physical fitness. Better physical fitness is generally related to a better health status, which in turn has a positive effect on being able to engage in physical exercise (Fig. 1). In this feedback loop, all arrows depict positive relationships. That means that the loop is a reinforcing one: if the value of one variable increases, this causes the others to increase as well, which in turn increases the first one again, and so on. However, if one of the three *decreases*, this causes the others (and itself) to decrease. A reinforcing feedback loop unaffected by other variables will tend to either keep increasing or decreasing. Feedback loops can also have a balancing character (a hypothetical example is found in Fig. 2), which means that the effects in the loop counteract each other so that they tend to fluctuate around an equilibrium.

### Scientific literature search

After the GMB sessions, in which a causal loop diagram was built, the relationships in this stakeholder model were further validated with evidence from the scientific body of literature. There is a substantial body of literature on the existence of a relationship between poverty and debt on one side and health on the other. However, checking the model means not only finding relevant literature on the relationships between these three variables, but on all relationships represented by each of the arrows present in the model. Doing a comprehensive literature review was therefore not feasible, since that would in essence involve conducting a full literature review for each individual relationship. For this reason, a quick scan of available scientific literature was carried out for all variables that are part of at least one mechanism (and with that also at least one feedback loop) between poverty, debt, and health. Searches were conducted with the use of Google Scholar and for each relationship in the stakeholder model, the names of the two connected variables were entered as search terms. For relationships for which this yielded few relevant results, variations of the search terms were also entered. Where necessary, additional databases – mainly Web of Science and PubMed – were used in order to obtain full-text versions of discovered literature. All studies that were identified in the Google Scholar searches as being potentially relevant, were retrieved. Literature was included if it makes any claim about the relationship between two connected variables in the model.

### Scientific meeting

To validate the outcomes of the GMB sessions as well as the outcome of the quick scan and to strengthen the model, the CLD was presented and discussed in a scientific meeting, in which five researchers with expertise on the relationships between social determinants of health and health participated. Whereas the stakeholders who took part in the GMB sessions were specifically selected for their experience in policy and practice, the researchers were selected for their familiarity with scientific knowledge on the topic. The scientific meeting was conducted in a systematic manner, in order to extract useful feedback [39] and it was recorded and afterwards transcribed ad verbatim. It consisted of three main parts, each with its own objective. The meeting was chaired by DR; LR, MJ and HH were also present in supporting roles.

The goal of the first part of the meeting was to obtain an assessment of the applicability of the model, as constructed by the stakeholders who participated in the GMB sessions, in a scientific context. Applicability here can be understood as the suitability of the model:

- As a conceptual model
- For analysing relationships between variables and analysing mechanisms
- For analysing possible impacts, such as health impact, that activities may have

The second part of the meeting focused on taking stock of any specific changes that would be necessary in order for the model to be in line with scientific knowledge. These could be additions, but could also be alterations or deletions of the existing arrows.

The third and final part of the scientific meeting was a discussion on the mechanisms present in the model – both from poverty and debt to health and vice versa – and so trying to identify core elements of the model. Such a core model would be less unwieldy and could offer a more focused starting point for the construction of a quantified model in the future.

## Results

### The stakeholder model resulting from the GMB process

**Fig. 3:**
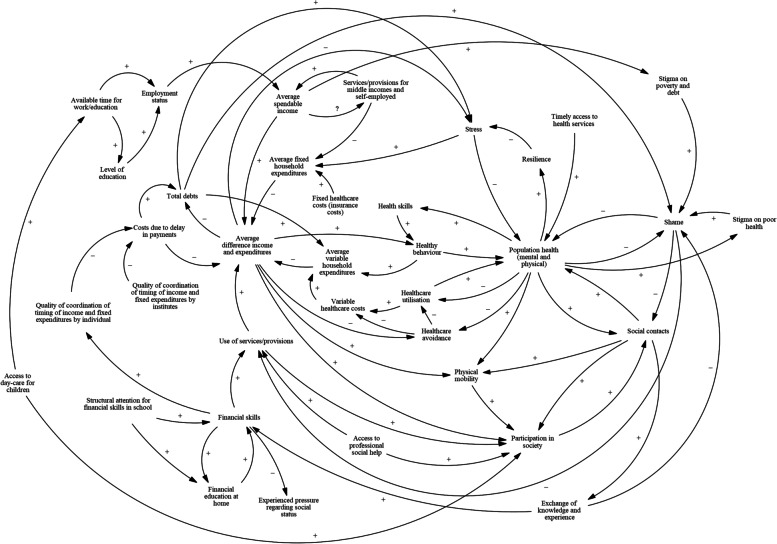
Causal loop diagram as constructed by stakeholders in Utrecht (Vensim, PLE version 9.0.1)

The result of the three stakeholder sessions is a CLD (Fig. 3). The model contains 39 variables and 71 causal arrows between those variables. Of these variables, 29 are in at least one feedback loop and of those, 26 are in at least one feedback loop with more than two variables. Most of these variables are involved in many loops, for instance: the variable of *total debts* is present in 117 different feedback loops (as counted by the modelling software); *population health* and *average difference income and expenditures* are both in 193 loops.

**Fig. 4:**
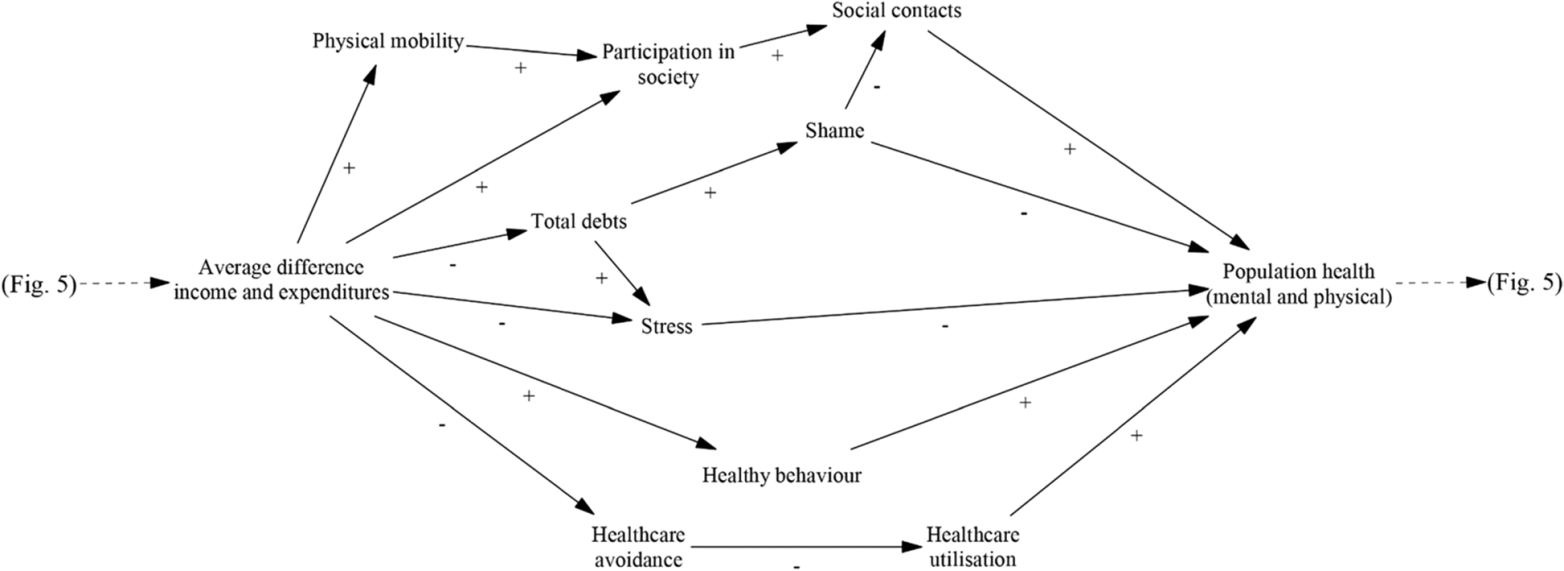
Mechanisms in the model leading from poverty and debt to health (Vensim, PLE version 8.2.1)

**Fig. 5:**
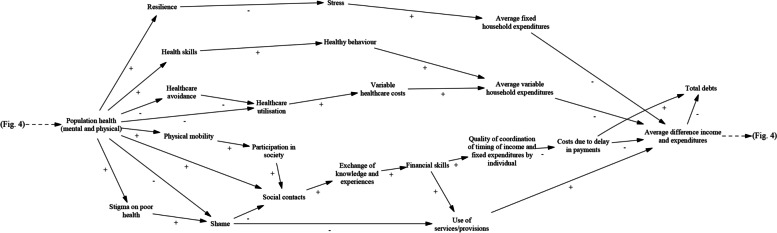
Mechanisms in the model leading from health to poverty and debt (Vensim, PLE version 8.2.1)

In an effort to display the mechanisms in the model in a somewhat more straightforward manner, Figs. 4 and 5 show the different pathways from poverty and debt to health and those from health to poverty and debt. These show eight causal pathways from *average difference income and expenditures* to *population health* and fourteen pathways from *population health* to *total debts*, of which eleven go through the variable of *average difference income and expenditures.* The variables of *participation in society*, *stress*, *shame*, *social contacts* and *use of services/provisions* are present in many mechanisms and seem to hold prominent roles.

The starting point for the model was made in the first GMB session, when the stakeholders identified and selected four central variables. They found these were *population health (both mental and physical), expenditures* versus *income*, *financial skills*, and *participation in society*. Poverty (implicitly present in *expenditures* versus *income*) and health had already been given as part of the overall topic for the GMB sessions by the facilitators, but there was no reason to specify that participants were not allowed to also name those variables as being at the heart of the issue themselves. Interestingly, the variable of *total debts* was added to the model structure as late as the third (and final) GMB session.

The four central variables warrant a brief description, which is given here. A full list containing brief descriptions of all the variables that are present in the model can be found in Additional file 3. Health is included in a very general way, as ‘mental and physical population health’. For practical purposes, the participants found it useful to keep the variable of health broad and to make separate variables in the model for *stress*, *shame*, *physical mobility*, *resilience*, *social contacts* and *participation in society*. Besides these specific health aspects that were explicitly included in other variables, the variable of *population health* can be understood as entailing well-being in a broad sense. Expenditures were formulated as all expenses, both fixed and variable, that a household makes in a given period of time; income as the amount of money (after taxes, before any expenses) coming in in the same period of time. *Financial skills* entail the skills that are necessary to understand personal finances, to keep an overview, and to make good financial choices. This does not necessarily have to mean that these skills are used to actually make such good financial choices. The participating stakeholders described *participation in society* in a broad and open way. It was taken to mean one’s sense of being part of society and not being excluded from important aspects of life: having a useful and fulfilling way of spending the day, and being able to participate in social activities.

The model both contains variables that are aggregated but operate mostly on the individual level, and variables that operate on a collective (meso or macro) level. Most variables are of the first sort: for example, healthcare avoidance or utilisation is ultimately done by individuals. There are some variables that are undeniably structural determinants, but these are not present in the model’s feedback loops. *Quality of coordination of timing of income and fixed expenditures by institutes* (such as the time in the month when citizens receive their tax allowances, in conjunction with when payments for fixed expenses are usually due), *services/provisions for middle incomes and self-employed*, *timely access to health services*, *access to professional social help*, and *structural attention for financial skills in school* are clear examples. Another structural variable in the model is *access to day-care for children*. In the third GMB session, the stakeholders reasoned that accessible day-care for children would allow parents to spend more time on work or on an education and to better participate in society. At the same time, there are no mechanisms in the model that can make day-care more accessible – that influence this structural variable. Arguing the need for regulation to bring about affordable and therefore accessible day-care, one participant concluded: *“( …*) *But that is a political thing, we can’t just change that”*. This is a good illustration of the stakeholders’ stance towards the structural issue, showing understanding of the specific problem and of a possible solution, but also a belief that structural change is beyond their means.

The anonymous evaluation form was completed by nine of the participants after the third session and showed general satisfaction with the CLD, the GMB sessions, and with what they learned from the sessions. All nine respondents expressed that they agreed or strongly agreed with the final CLD. Six of them reported an increase in insight into the topic and eight participants stated their understanding of the way the issues are interconnected improved as a result of the sessions. Seven of them agreed or strongly agreed with the statement that they were able to apply the model to practical situations themselves and also indicated that they expected to do so in the future.

### Validation of the stakeholder model through scientific literature

As mentioned in the methods section of this paper, the stakeholder model was corroborated with evidence from scientific literature, to see if there was scientific evidence on the individual arrows in the model. Literature on these relationships that was not in line with stakeholders’ expectation was also included. For some of the relationships, a great deal of evidence was found; for others, less so. The former is an indication that the relationships is grounded in scientific knowledge, but the latter does not necessarily indicate that the relationship does not exist. Evidence for the opposite of a modelled effect (negative instead of positive or vice versa) does however argue that the model may benefit from adjustment. A total of 70 articles were found and included as evidence supporting or contradicting the relationships in the model. Studies on the relationships involving population health, stress, shame, social contacts, and healthy behaviour were generally easy to find. The variables regarding stigma, participation in society, financial skills, resilience, delays in payments, and services and provisions for middle incomes and self-employed seem to be less well-represented in the literature. A full overview of the results of the literature scan can be found in Additional file 4.

One relationship that was formulated by the participating stakeholders was the difference between income and expenditures, and debts. The expectation was that if income minus expenditures gets lower, total debts would increase. However, scientific evidence shows that households that are financially better off and more stable tend to have higher levels of debt [40, 41]. A possible explanation for this is relatively straightforward: These households should be able to safely get higher loans, such as mortgages, and therefore do take on higher debts. Conversely, lower-income households on average have less debt in absolute terms, but have more difficulty making the payments on their debts [41]. If one wants to study the relationships between poverty, debt, and health, it seems meaningful to focus specifically on *problematic* debts: cases in which there is a high ratio between burden of debt and income.

The relationship between resilience and stress is not as much contested in the literature that was found, but studies suggest that the relationship works differently. In the stakeholder model, health affects resilience, which in turn affects experienced stress. From the literature it appears that resilience may be influenced by health, but that it has a moderating effect on the relationship between health and stress, instead of a direct effect on stress [42, 43].

### Validation of the stakeholder model by scientific researchers

The participating researchers commented on the model as constructed by the stakeholders. In general, they supported the relationships that are present in the model and thought that the vast majority of these would be supported in scientific literature. Conversely, the consensus in the scientific meeting was that the most important solutions to the problems surrounding poverty and health are usually distal, structural determinants, while the model mostly contains individual-level mechanisms. Variables and mechanisms concerning housing costs, social security and re-integration provisions for people with health problems were mentioned as likely influential additions.

They also noted that some of the variables are very detailed and almost fully operationalised, while others – such as *population health* or *participation in society* – are still quite vaguely formulated. Especially the variable containing health could benefit from further elaboration; different effects for different kinds of health could be expected.

Additionally, some relationships that the participating researchers would expect to be present, were not. *Employment status* and *level of education* were deemed to have a much more central role than the model gives them credit for and *participation in society* was considered to affect income. Furthermore, *stress* was expected to influence *health behaviour* and *shame* to influence *healthcare avoidance*. Also, the researchers would have expected to see some interaction effects in the model.

## Discussion

This paper addresses the question how group model building can contribute to gaining insight into a wicked problem concerning social determinants of health. The approach is demonstrated by taking poverty, debt, and health in Utrecht as a case study. The resulting model is almost by definition a valid representation of the way the participating stakeholders experience the topic in practice. In this study, a qualitative model of how poverty, debt, and health are related to each other was built. It was not the study’s aim to directly to link specific existing or proposed interventions to the model in order to ascertain their expected effects or to find solutions to the problems. However, the latter can be done by simply following the arrows that flow from any variable that is believed to be directly affected by an intervention, though a model such as a CLD does not contain any expectations about effect sizes.

The results section of this article and the final CLD (Fig. 3) show a large number of possible links between poverty, debt, and health and each of these are present in many feedback loops. This means that any change in one of these variables is anticipated to have a large number of direct and indirect effects, including on itself. *Participation in society*, *stress*, *shame*, *use of services/provisions*, and *social contacts* are also present in many mechanisms and could be seen as ‘central nodes’ in the model, which implies that they are important variables in the system. It is important to note that this is not necessarily so. A variable that is only present in one mechanism may potentially exert a strong influence through it, while another variable might produce only weak effects in a lot of mechanisms. The number of mechanisms a variable is present in does however give an indication that including the variables mentioned above in an integral approach regarding poverty, debt, and health is more likely to be particularly beneficial.

The results from the case used in this study also show that the stakeholders are well able to construct a model of such a wicked problem and that its complexities are understood by the stakeholders. The model-building process demonstrates that explicitly formulating such a model brings added value by itself: the stakeholders who have constructed the model will not only be aware of the complex mechanisms in it, but also have model ‘ownership’. This makes it more likely that they will accept, share, and utilise it in agenda setting, policy formulation, and policy implementation [44]. After the GMB sessions conducted in this study, participants indeed indicated that they were able to use the model and expected to do so in the future. This at least suggests that the model can potentially be used in practice. Doing so seems beneficial: due to feedback loops, there is little chance that the entire set of interrelationships that are present in the model works in a fully intuitive way with linear mechanisms [45]. For this reason, any intervention that one would implement in practice is likely to produce at least some consequences that would ordinarily be unforeseen – consequences that stakeholders may be able to anticipate, using such a model. The model also illustrates how variables are reciprocally connected and that an integral approach to the problem may be beneficial.

Both the stakeholders who participated in the GMB sessions and the scientific researchers contributing to the validation check of the model noted the importance of structural determinants, such as education, labour, housing costs and coordination by institutions. The stakeholders did appear to regard changing these systemic conditions and therefore using them as structural *solutions* as more difficult, resulting in these determinants to not be included in the model’s feedback loops. This indicates that a model for solutions to structural problems may benefit from being expanded – the results from the GMB show that interplay between individual and structural social determinants of poverty, debt, and health on all levels may seem limited to stakeholders embedded in practice. In other words, stakeholders are aware of structural factors that play a role of importance, but seem to consider them to be beyond their control and find it difficult to point out how they could be affected. The researchers who reviewed the model in the scientific meeting also stressed the importance of focusing on such structural variables in order to effect significant change. This study identified some variables, such as access to day-care for children and the timing of payment by institutions such as tax and welfare agencies, in which structural improvements could potentially be made. In order to do so, it is important to reach and include actors who have the ability to implement changes that are necessary for that.

Another important point brought up by the scientific researchers is that additional tests of the model using quantitative data should be considered in order to further validate the conceptual model for scientific use and that some adjustments of the model structure may be appropriate for this purpose. Such tests have the additional benefit that effect sizes of individual relationships could also be estimated in this way.

This study has demonstrated an approach using three different sources of evidence: participation both through GMB sessions and a scientific meeting, complemented with scientific literature. Using the participatory method of GMB provides one with a way to obtain a qualitative model– and crucially, a demarcation – of the issue in a structured, non-arbitrary way. There is also added value in having stakeholders from the local community who identify the problems themselves, rather than identifying problems from outside [46]. The sort of experiential understanding that local stakeholders can provide will often not be available from other sources. Subsequent examination of these findings by means of scientific literature and a scientific meeting helps identify the model’s strengths and potential caveats. The nature of information from scientific literature is very different from stakeholders’ input. Findings in this literature are the results of often extensive and rigorous studies, which use empirical data to make claims about the world. Also, their scopes are much narrower, focusing on just several variables of interest. The meeting with scientific researchers could be regarded as eliciting knowledge that is experiential and different from the local stakeholders. They do not have the same practical experiences that the local stakeholders have, but their knowledge instead has a basis in scientific evidence. Their type of knowledge can therefore be regarded as having similarities to both of the other types of evidence, while being different. Utilising these three types of information, each with a different purpose and value, offers a more nuanced view than only one or even two of them would have [47, 48].

The methodological approach described in this paper is generalisable and can be applied to other contexts and to the wicked problems constituted by other social determinants of health as well. As to the resulting model in this study, one should keep in mind that this model is not *the* model of the relationships between poverty, debt and health, but rather *a* model of a specific situation in a specific context. The model for the particular wicked problem selected for this study was made specifically for the city of Utrecht. It may be that the model for Utrecht is also applicable to other settings, but the wickedness of the problem entails that it cannot simply be assumed to apply to other settings or situations [8] – context should always be taken into account. In a CLD that is built using GMB, contextual factors are usually not at the centre of the model, due to the method’s focus on feedback mechanisms. A researcher aiming to apply such a model in other contexts would therefore have to identify and compare relevant contextual factors themselves.

In a previous study, a similar approach with GMB has been used to model the interrelationships between poverty, chronic stress and health in the Dutch city of The Hague [16]. The model resulting from that study was different from – but not in contradiction with – the model presenting in this article. Notably, the variable of health was split into life expectation, burden of disease, fitness and emotional wellbeing, while the financial side of the model (income, expenditures, debts) was described in less detail. Such differences are expected and can be attributed to the different context and to the fact that other stakeholders participated in this different setting – the two of which cannot be seen as completely separate. The authors of the The Hague study also recognise the influence both of the context of the study and of which stakeholders participate.

The complex and indirect influences of social determinants of health, together with that they often have long delays in producing health outcomes, make them difficult to study [49]. This may inhibit researchers from studying such topics and thus producing evidence on their effects. Evidence, in turn, is an essential requirement for policy action [50]. A lack of evidence concerning social, and in particular structural or systemic, determinants of health may result in them having diminished emphasis and attention in policy and practice. At the same time, the magnitude of their effects [1, 2] makes it essential that they do receive proper attention. Determining what is on the policy agenda is a very important part of the process that decides which (kinds of) issues are dealt with and which are not and in which terms these issues are discussed [51, 52]. We hope that the approach used in this study helps social determinants of health get the necessary attention, by showing a way in which their effects can be understood.

A recommendation that follows from this study, in line with the recommendation of the scientific researchers, is that a model that is built using this approach can be used as a conceptual model for quantitative analysis. In this way, the relationships that were expected by participants can be tested – providing stronger evidence – and the resulting quantitative model can also be used for estimating relationships strengths and impact sizes. Depending on the specific topic and the nature of the relationships in the model, it is likely that micro-level, longitudinal data will be needed for this. A combination of GMB using system dynamics (as shown in this study) and a different, micro-level estimation method can be used and would be appropriate in such an endeavour [9]. It should be stressed that a system works as a whole and should be regarded and quantified as such, although some simplifications to a full CLD are likely to be necessary for quantification. The individual relationships in the model should preferably not be quantified in separate models, due to multicausality and overlapping effects [53]. However, there are modelling methods that can deal with a system of multiple outcome variables with reciprocal effects [54, 55]. There are also some clear limitations to this study. One of the strengths of the approach is the involvement of stakeholders, but this at the same time produces some additional considerations. As mentioned, we decided to select a group of stakeholders who were involved with the issues mostly in the practical field, with the intention of obtaining a model that considers the topic from a practical point of view. As a result, it focuses on how variables are likely to affect each other on an individual level, rather than what causes changes in variables on a structural level. Many structural factors are likely to have far-reaching effects, but they are also external to this specific model. Other researchers might have selected different participants, who would have constructed a different model reflecting a somewhat different perspective, not necessarily due to a difference in the context in which they are situated [7, 19]. This subjectivity is inherent in this type of qualitative research and unavoidable. The system models that result from the GMB process consist of hypothesised relationships between variables and can and should be subsequently scrutinised, as done in and recommended by this study.

## Conclusions

In conclusion, a causal loop diagram such as presented in this paper displays how stakeholders experience reality, what they see as important problems and which potential solutions they see as being within their influence and which ones they do not. Also strengthened by validation checks using the scientific literature and consulting scientific experts, the model adequately works as a strong hypothesis-generating fundament: it provides a good starting point for showing what a complex reality may look like. This can help to anticipate effects that may follow from (a mix of) interventions. Additionally and importantly, it can be used as input for quantitative modelling. The saying “All models are wrong but some are useful” ([56], p.2) applies here.

## Supplementary Information

**Additional file 1.**

**Additional file 2.**

**Additional file 3.**

**Additional file 4.**

## Data Availability

Most of the results presented in this study are based on participation. Raw data based on this participation (such as recordings or transcripts) cannot be made available, due to privacy and consent. Other requests for additional information can be addressed to corresponding author. The software used to create the models in this study (Figs. 1-5) is Vensim PLE versions 8.2.1 and 9.0.1 by Ventana Systems, which is available for Windows and Macintosh OS (X) from https://vensim.com. This version of the software is free for academic use, but needs a paid license for non-academic use.

## Abbreviations

CLD: Causal loop diagram
GMB: Group model building
SDoH: Social determinant(s) of health
