# Supplementary material for: Qualitative modelling of social determinants of health using group model building: the case of debt, poverty, and health
Source: Int J Equity Health. 2022 May 19;21:72. doi: 10.1186/s12939-022-01676-7 (PMC9118602; doi:10.1186/s12939-022-01676-7)
Supplement: Supplementary file 1 — Additional file 1. [file 12939_2022_1676_MOESM1_ESM.docx]

**Additional file 1: Guide for semi-structured interviews with stakeholders in preparation for the GMB sessions**

*Length: 15-25 minutes*

1. **Background information about the participant**

- Can you tell me some things about your position at [organisation]?
  - What are the main aims and activities of [organisation]?
  - What is your position at [organisation]?
  - How long have you been working at [organization]?
  - What was your primary motivation for doing this job?
  - In your current position, how are you involved with:
    - Poverty;
    - Debt;
    - Health?
  - Have you been involved with any of these topics in other/previous positions?
- Can you name some of the main partner with whom you are cooperating on the topic(s) of poverty, debt, and/or health?
  - Are there other important parties regarding these topics within the city of Utrecht that you are familiar with, but are not important partners for you directly?
- I will not ask any further questions on this matter, but do you see people outside of your professional position who are dealing with issues on these topics – and if so, which of the three (poverty, debt, health)?

1. **Participant’s vision towards the problems and initiatives that may counter them**

- Looking at poverty and debt in the city of Utrecht, what do you think some of the biggest problems are?
  - Do you think that activities are being conducted that counter these problems?
    - [If yes] Could you name some of these activities?
- Looking at health in the city of Utrecht, what do you think some of the biggest problems are?
  - Do you think that activities are being conducted that counter these problems?
    - [If yes] Could you name some of these activities?

1. **Expectations regarding the GMB sessions**

- You probably have some kind of global expectations about what we are going to do during the coming sessions.
  - Can you tell me something about these expectations?
    - The sessions will be designed according to a group model building format. Do you happen to be familiar with this method?
      - [If yes] What is your experience with the method?
    - Do you happen to be familiar with system dynamics?
      - [If yes] What is your experience with the method?
  - What has lead you to agree to participate in the sessions?
    - [If necessary] Is your motivation to participate mainly because you want to attain new insights, or help further general knowledge concerning the topic, or a bit of both?
  - What results do you expect the sessions to yield?
