# Supplementary material for: Qualitative modelling of social determinants of health using group model building: the case of debt, poverty, and health
Source: Int J Equity Health. 2022 May 19;21:72. doi: 10.1186/s12939-022-01676-7 (PMC9118602; doi:10.1186/s12939-022-01676-7)
Supplement: Supplementary file 2 — Additional file 2. [file 12939_2022_1676_MOESM2_ESM.docx]

**Additional file 2: List of variable descriptions**

- **Access to day-care for children:** Accessibility (including cost barriers) of day-care for children.
- **Access to professional social help:** The accessibility of professional help that can help resolve the sorts of issues that can make people socially isolated and/or placed outside of society.
- **Available time for work/education:** The amount of time that one can spend on one’s work or education.
- **Average difference income and expenditures:** Spendable household income minus fixed and variable expenditures.
- **Average fixed household expenditures:** Household expenditures that are fixed for a (often long-term) period of time. For example, housing costs are usually significant fixed household expenditures.
- **Average spendable income:** Household income after income taxes.
- **Average variable household expenditures:** Household expenditures that may vary according to short-term budget choices and do not include long-standing engagements. For example, expenditures for food and entertainment are variable expenditures.
- **Costs due to delay in payments:** Additional costs that result from delay in paying bills, such as fines and debt collection costs.
- **Employment status:** Whether one is employed or not.
- **Exchange of knowledge and experiences:** Talking about personal problems with one’s social contacts, offering and/or hearing about experiences.
- **Experienced pressure regarding social status:** The social pressure that is felt by individuals to do certain unnecessary and unwise purchases. An example that was given is that of mobile telephones: in some groups, having the latest telephone brings social status and a sense of belonging, even if one is realistically unable to pay for it.
- **Financial education at home:** The quantity and quality of attention for financial education at home.
- **Financial skills:** The skills required to understand and navigate the financial landscape, to maintain an overview of personal finances and to make responsible financial decisions.
- **Fixed healthcare costs (insurance costs):** Fixed healthcare expenditures – defined here as insurance costs – that households have to pay. These insurance costs may vary due to different available packages.
- **Healthcare avoidance:** Not making use of healthcare in situations in which it is clear that healthcare is needed.
- **Healthcare utilisation:** Making use of health care, including mental health care.
- **Healthy behaviour:** Behaviour that related to lifestyle factors, such as food choices, physical exercise, alcohol, smoking and drugs.
- **Health skills:** The knowledge and skills that are needed for a person to exhibit healthy behaviour. This does not necessarily mean that the presence of these skills ensures someone to actually exhibits healthy behaviour. The stakeholders described this variable as a necessary condition but not a sufficient condition for healthy behaviour.
- **Level of education:** level of education attained by individual persons.
- **Participation in society:** A variable that was defined as being broad and rather open, as participation in societal and social activities. Part of this can be described as feeling that one is not situated outside of society and/or is unable to participate in important aspects of life. An element of this could be a meaningful and satisfactory way of spending one’s daily life.
- **Physical mobility:** The physical mobility of a person in terms of how well they are able to move around from one location to another. This includes both physical ability (how well is someone able to physically move) and other means, such as owning a car or having money for other modes of transport.
- **Population health (mental and physical):** A target population’s health, taken as well-being in a broad sense. Several other variables in the model could also be regarded as being part of health, such as stress, shame, resilience, participation in society, physical mobility, and social contacts. However, those are externalised in the model, largely for practical purposes, and therefore treated as separate from health.
- **Quality of coordination of timing of income and fixed expenditures by individual:** How well individuals coordinate their households’ incoming and outgoing money flows and manage the timing for the two – making sure income comes before expenses.
- **Quality of coordination of timing of income and fixed expenditures by institutes:** How well institutes responsible for paying out sources of households’ income and/or responsible for collecting fixed household expenses coordinate the timing of two – making sure income comes before expenses.
- **Resilience:** The ability to deal with and overcome difficult situations and adversity.
- **Services/provisions for middle incomes and self-employed:** The existence of financial and social services and provisions for middle incomes, including those who are self-employed. This entails persons who are not eligible to receive benefits such as public housing, rent allowance, or healthcare allowance, but are not so well-off financially that they can prosper without these benefits.
- **Shame:** The feeling of shame in broad terms: the fear of not being accepted, in any way or for whatever reason.
- **Social contacts:** The quality of a person’s social network. Whether someone has other people to talk with and engage in social activities with.
- **Stigma on poor health:** The way in which a group collectively looks at having poor health. A stigma can be said to exist if this is done in a judgmental manner. The extent to which a stigma is present, can also vary.
- **Stigma on poverty and debt:** The way in which a group collectively looks at being in poverty and/or debt. A stigma can be said to exist if this is done in a judgmental manner. The extent to which a stigma is present, can also vary.
- **Stress:** An umbrella term of all different forms of acute and chronic stress. The stakeholders argued that the model did not necessitate different mechanisms for different forms of stress.
- **Structural attention for financial skills in school:** The quantity and quality of structural attention that schools dedicate to financial education.
- **Timely access to health services:** Whether people are able to access health services in time, from a collective, systemic point of view. Health issues (both mental and physical) can grow over time if left untreated, making them more difficult to treat in the end.
- **Total debts:** Average total debts held by households. This includes all kinds of debt.
- **Use of services/provisions:** Making use of existing public services and/or provisions. There are often provisions for those who are in need of them, but they are not always found or correctly applied for and utilised. For example, applying for benefits or allowances requires knowledge of their existence and a certain degree of initiative to get them.
- **Variable healthcare costs:** All healthcare expenditures that are not covered by health insurance.
