# Supplementary material for: Qualitative modelling of social determinants of health using group model building: the case of debt, poverty, and health
Source: Int J Equity Health. 2022 May 19;21:72. doi: 10.1186/s12939-022-01676-7 (PMC9118602; doi:10.1186/s12939-022-01676-7)
Supplement: Supplementary file 3 — Additional file 3. [file 12939_2022_1676_MOESM3_ESM.docx]

**Additional file 3: modelling steps taken during the group model building sessions**

***Colour coding***

The four figures in this supplement chronologically show the development of the causal loop diagram (CLD) as developed by the participating stakeholders. For clarity’s sake, the models in this supplement were giving colour codes that are different from those in the main text of the article. Whereas the models in the main text use blue arrows for positive relationships and red arrows for negative relationships, the colours in this supplement indicate additions and adjustments compared to the model’s previous version. Variable names in black and arrows in blue indicate that they have not been changed, variables and arrows in red are new in that modelling step and green variables were already present but had their names altered.

***Figure 1***

The central building blocks of the causal loop diagram are the four ‘central’ variables that were selected by the stakeholders in the first group model building (GMB) session. These were *(mental) health*, *income versus expenditures*, *financial skills* and *participation in society*. *Income versus expenditures* was recognised to consist of at least two variables and was split into the variables of *spendable* *income* and *fixed expenditures,* but the combined variable was also kept in the variable of *difference income and expenditures*.

Other variables from the list of variables that was collected in the first session were added to the model structure. In order to do so, the stakeholders were asked to select from this list causes and consequences of the five central variables. Some additional variables (not from the list made in the first session) were also included, where the stakeholders deemed this necessary. The question mark accompanying the arrow from *spendable income* to *services/provisions for middle incomes and self-employed* points out that the stakeholders did not reach consensus on whether this relationship would be a positive or negative one.

***Figure 2***

In this step, the four separate structures from Figure 1 were connected to each other. This was achieved by adding a number of relationships. The stakeholders were given the task to connect the central variables to the other ones without drawing any direct arrows between them. In doing so, they formulated mechanisms that tell *how* the different parts of the model are interconnected. The model in this figure was the result after GMB session 2.

***Figure 3***

In this relatively small edit, the variables of healthy behaviour and average variable household expenditures were added with the purpose of elaborating the mechanisms between health and *average difference income and expenditures*. The names of six variables were altered.

***Figure 4***

This is the resulting model as it was made in the third and final GMB session. Some variables were added, among which were *total debts*, *level of education* and *employment status*. Also, most of the structural variables were identified in this session. *Chronic stress* and *acute stress* were merged into simply *stress*, as the stakeholders argued that the location of the two types of stress would be the same in this model.


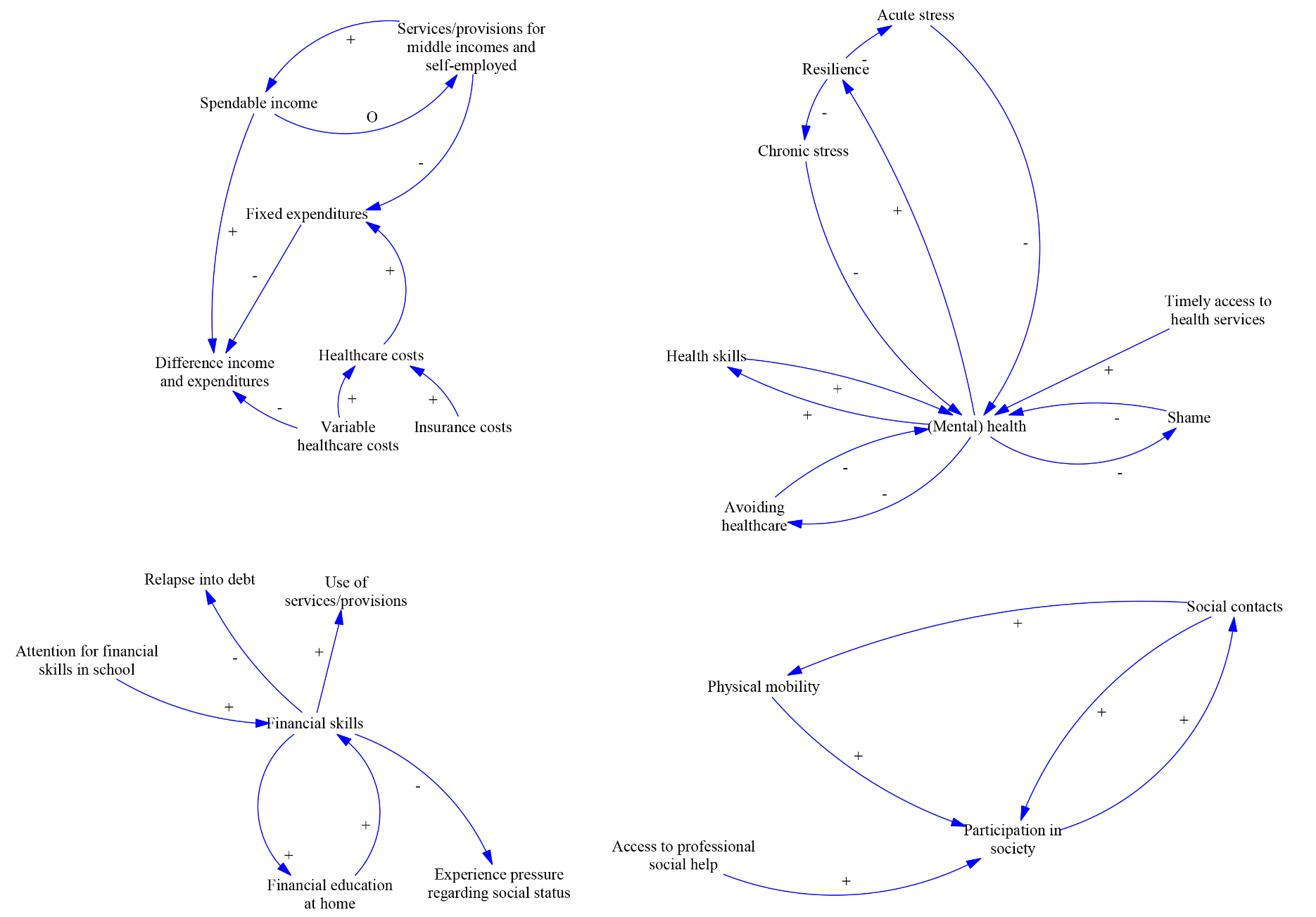


***Figure 1: separate model structures built around the central variables***


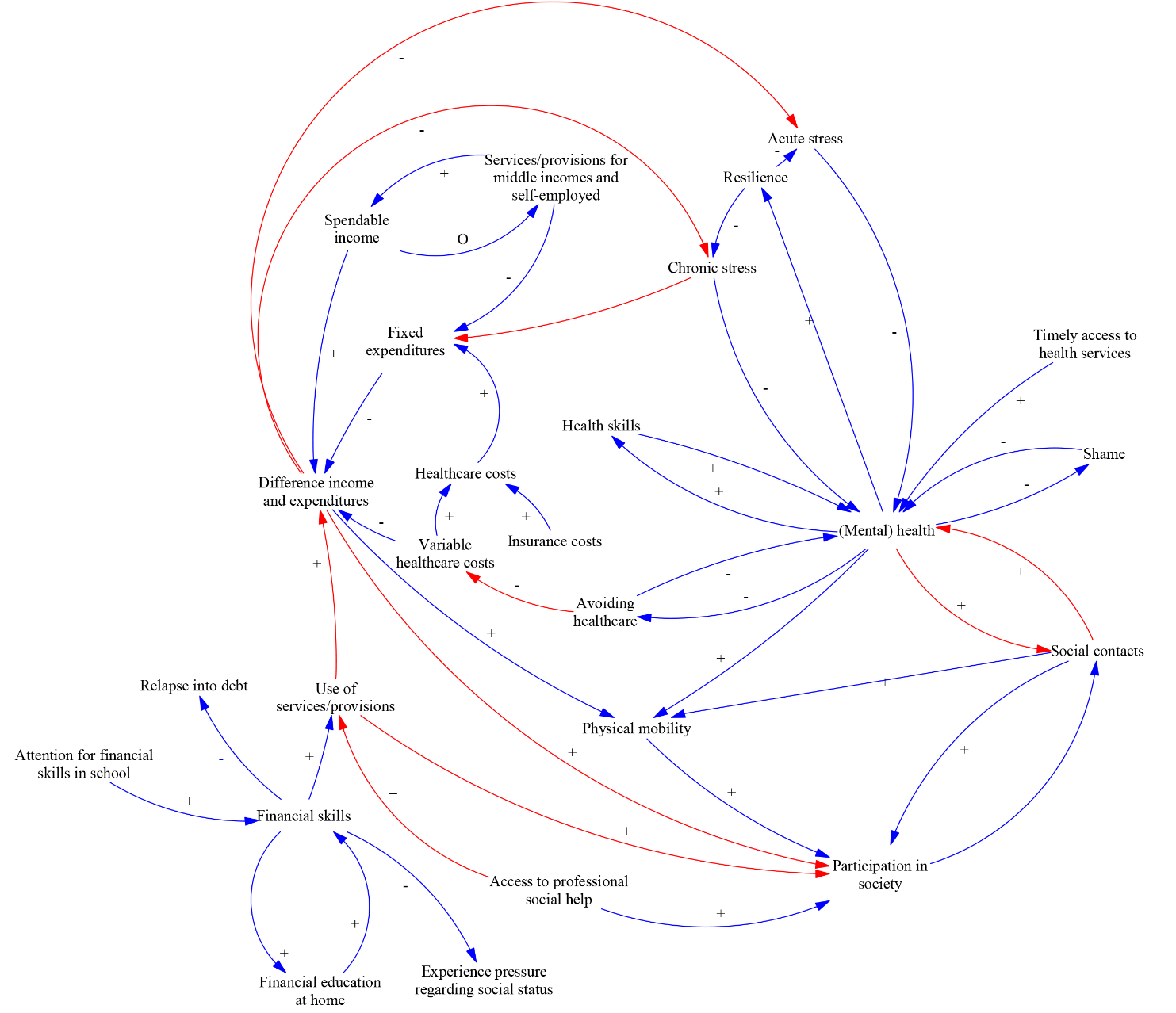


***Figure 2: the model after the separate parts were connected to each other***


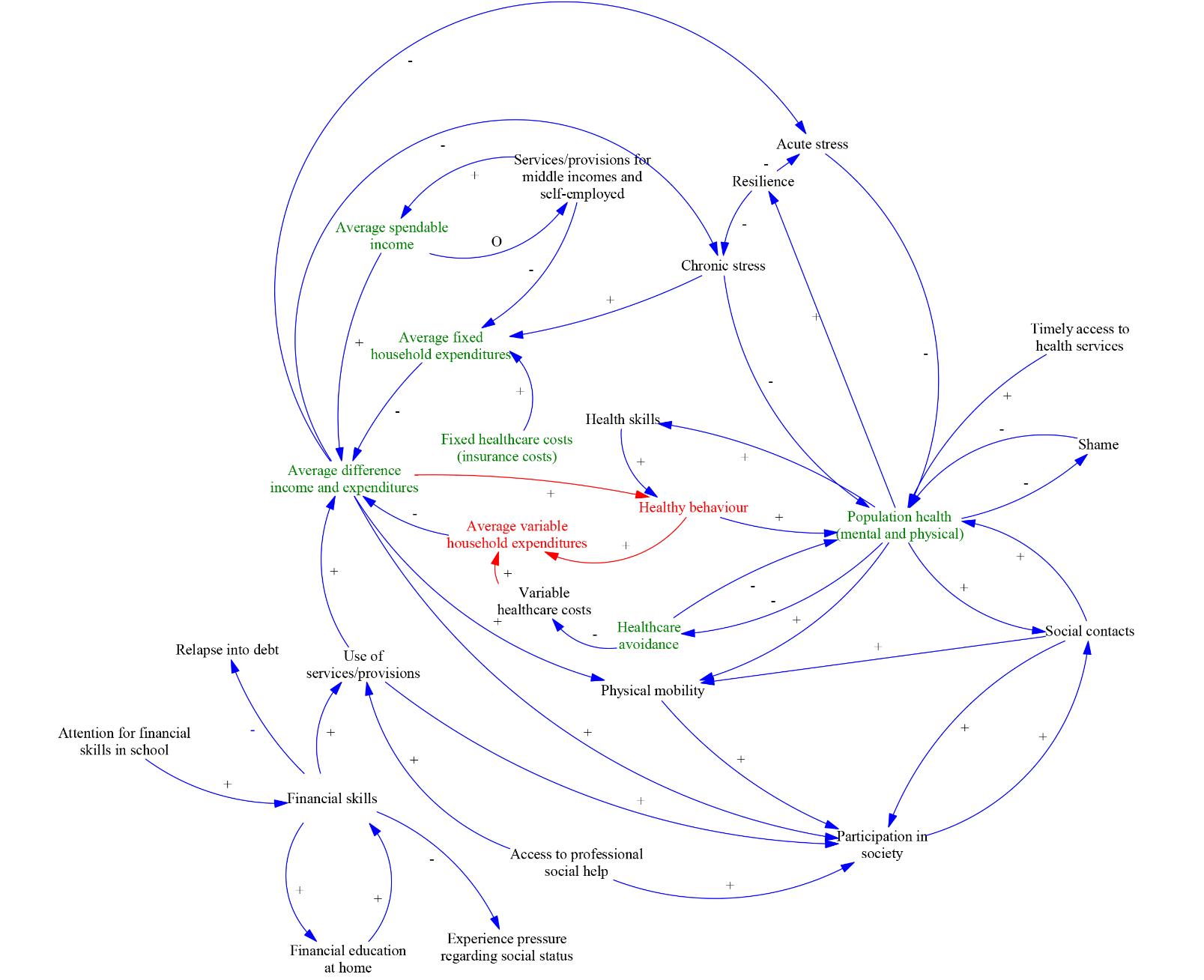


***Figure 3: model with some changes in variable names and an altered connection between the financial and health sides***


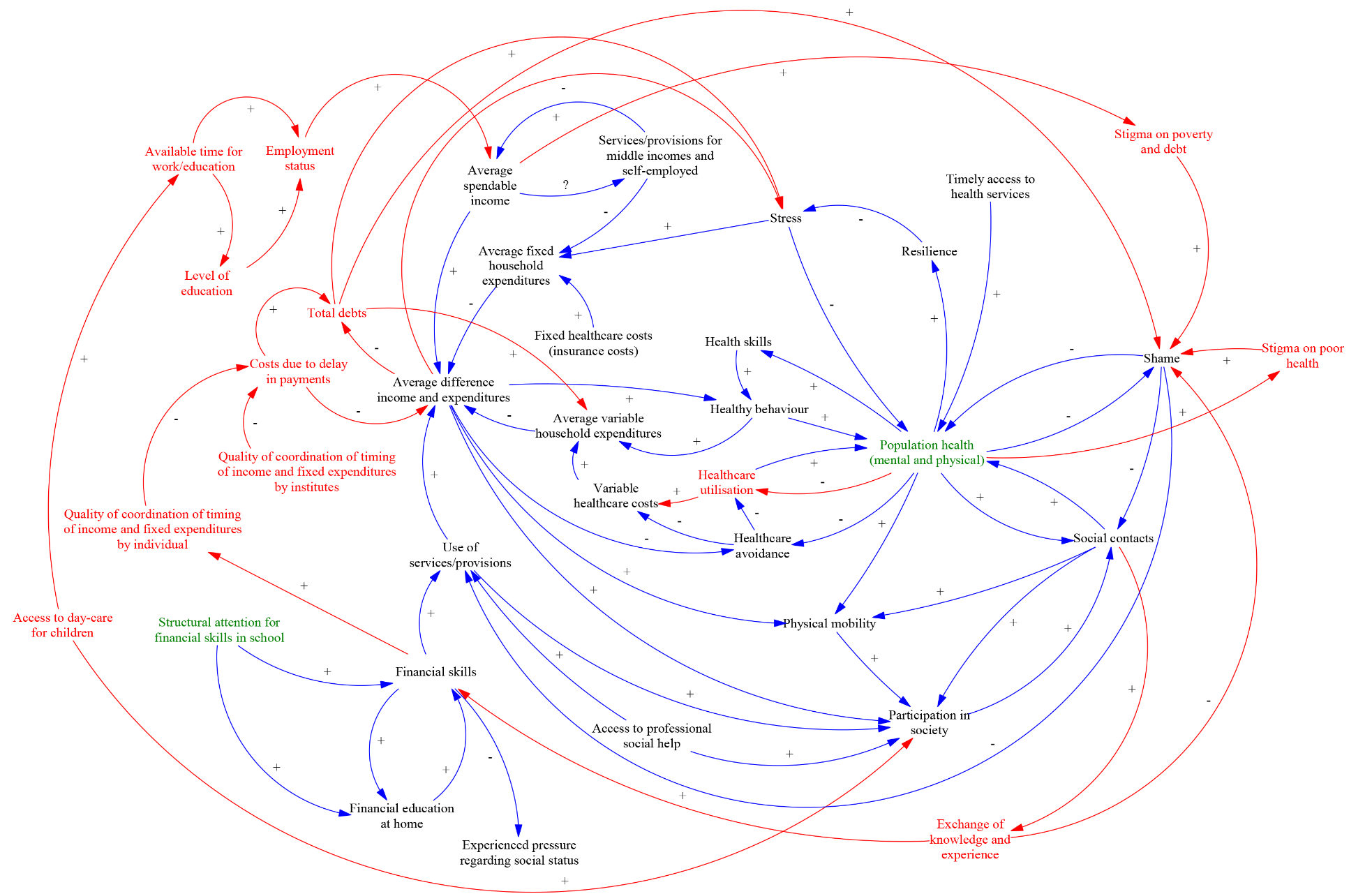


***Figure 4: expansion of the model with a number of additional variables, resulting in the final stakeholder model***
